# Age estimation using the radiographic visibility of the periodontal ligament in lower third molars in a Portuguese population

**DOI:** 10.4317/jced.51813

**Published:** 2014-12-01

**Authors:** Catarina-Dourado Sequeira, Alexandra Teixeira, Inês-Morais Caldas, Américo Afonso, Daniel Pérez-Mongiovi

**Affiliations:** 1 Faculdade de Medicina Dentária da Universidade do Porto. Porto, Portugal; 2 CENCIFOR - Center of Forensic Sciences, Portugal. Associação Centro de Ciências Forenses. Coimbra, Portugal

## Abstract

Objectives: The mineralization of third molars has been used repeatedly as a method of forensic age estimation. However, this procedure is of little use beyond age 18, especially to determinate if an individual is older than 21 years of age; thus, the development of new approaches is essential. The visibility of the periodontal ligament has been suggested for this purpose. The aim of this work was to determine the usefulness of this methodology in a Portuguese population. Study Design: Periodontal ligament visibility was assessed in the lower third molars, using a sample of 487 orthopantomograms, 228 of which belonging to females and 259 to males, from a Portuguese population aged 17 to 31 years. A classification of four stages based on the visual phenomenon of disappearance of the periodontal ligament of fully mineralized third molars was used. For each stage, median, variance, minimal and maximal age were assessed. Results: The relationship between age and stage of periodontal ligament had a statistical significance for both sexes. In this population, stage 3 can be used to state that a male person is over 21 years-old; for females, another marker should be used. Conclusions: This technique can be useful for determining age over 21, particularly in males. Differences between studies are evident, suggesting that specific population standards should be used when applying this technique.

** Key words:**Forensic sciences, forensic odontology, age estimation, third molar, periodontal ligament.

## Introduction

Forensic age estimation has become increasingly important. This importance is related, partially, to problems arising from globalization, namely the increasing number of non-national subjects with doubtful information regarding their birth date. In these cases, age estimation is necessary in the course of criminal, civil or asylum proceedings (1-7). The international interdisciplinary Study Group on Forensic Age Diagnostics [Arbeitsge-meinschaft für Forensische Altersdiagnostik (8)] has proposed some guidelines for forensic age estimation. These include: a] clinic examination, performing anthropometric measures and assessment of sexual maturity signs; b] x-ray examination of the left hand; c] dental evaluation with a clinical examination and analysis of an orthopantogram [OPT]. If the skeletal development of the hand is completed, an additional X-ray examination or CT scan of the clavicles should be performed (4,6,9-11). Often, the information needed relates with the probability of an individual being older than 14, 16, 18 or 21 years old (4,12,13); in these situations, third molars are the only teeth able to provide valuable information, since all the remaining teeth have finished their development process. However, third molar mineralization is frequently completed under age 21 (14,15) and, in some populations, it can be completed under age 18 (12,16,17). In the Portuguese population, a previous study has showed that stage H of the eight-grade Demirjian scheme was found to be an useful marker for diagnosing age equal or superior to 18 years, both in males and females (12). This means that for determining age equal or superior to 21 years, this methodology has no usefulness, since most teeth have already ended their root development by age 18. Several new dental techniques have been recently proposed to estimate age in these cases. For instances, Olze *et al.* (3) referred to the use of the periodontal ligament in the lower third molars as potential age estimation criterion after completed formation of the root.

In this study, the radiographic visibility of the periodontal ligament in third molar was analyzed using OPT from a Portuguese population, in order to determine the suitability of this methodology in forensic age assessment, especially for determine age over 21 years.

## Material and Methods

A total sample of 487 OPTs from Portuguese subjects from ages 17 to 31 years were assessed; 228 belonged to females, and 259 to males; subjects were divided into two groups: group a]: age inferior to 21 years; group b]: age equal or superior to 21 years. Subjects attended the residency dental clinic of the Faculty of Dental Medicine of University of Porto. OPTs were taken for diagnostic purposes, and dates of birth and exposure were proven, but unknown to the examiners. The socioeconomic background of the subjects’ sample can be described as middle to low and the general health status of the sample subjects was good, with no known systemic pathologies. The population affinity could not be verified however all subjects had Portuguese last names and it can be assumed they were born and lived in Portugal. This procedure was adopted by other authors in similar circumstances (3,12,18).

All OPTs with unclear images or presenting impacted third lower molars or with incomplete root formation, carious lesions or endodontic treatment were excluded. Age and sex distribution of the material can be observed in  Table 1.

**Table 1:**
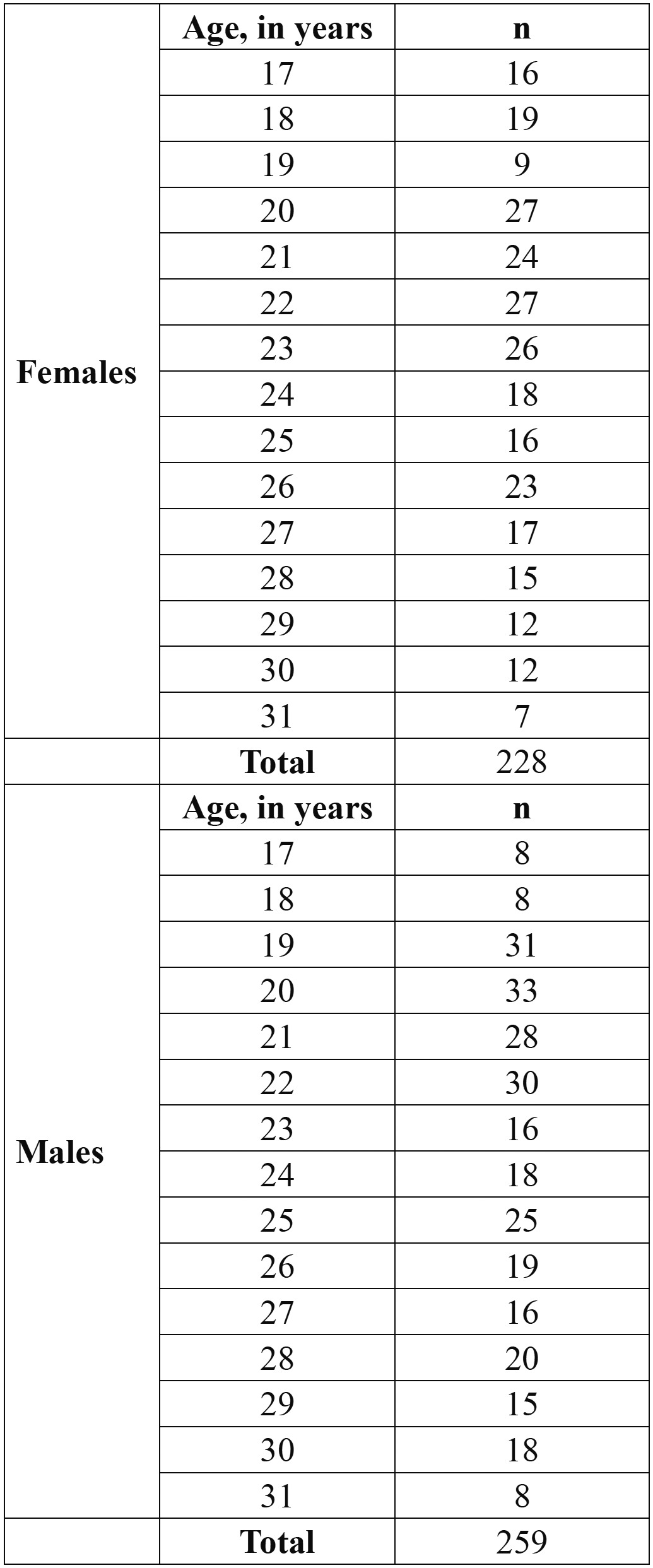
Age distribuition.

**Figure 1:**
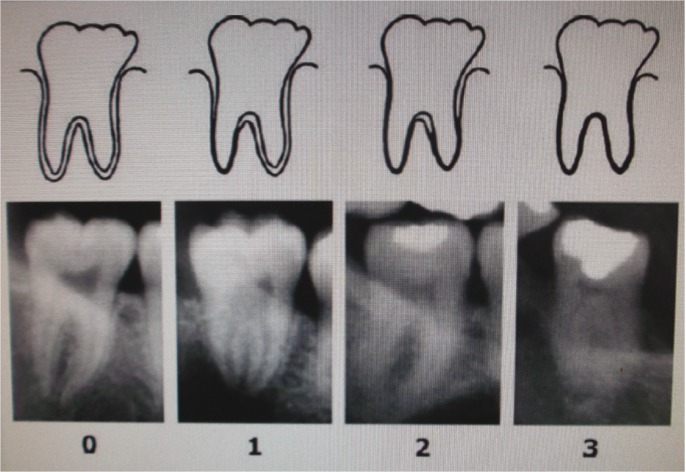
Schematic drawings and pictures of stages of radiographic visibility of the periodontal ligament in lower molars (adapted from: Olze A, Solheim T, Schulz R, Kupfer M, Pfeiffer H, Schmeling A. Assessment of the radiographic visibility of the periodontal ligament in the lower third molars for the purpose of forensic age estimation in living individuals. Int J Legal Med 2010; 124(5):445-8).

The visibility of the periodontal ligament of lower third molars with completed root formation [including apical closure] was recorded, as defined by Olze *et al.* (3) in the following four stages (Fig. 1):

- Stage 0: The periodontal ligament is visible along the full length of all roots;

- Stage 1: The periodontal ligament is invisible in one root from apex to more than half root;

- Stage 2: The periodontal ligament is invisible along almost the full length of one root or along part of the root in two roots or both;

- Stage 3: The periodontal ligament is invisible along almost the full length of two roots.

As recommended by Olze *et al.* (3), stage 3 was not applied to third molars with only one root. The periodontal ligament was assessed in the left lower third molar [n 38] and, when this tooth presented any of the exclusion criteria referred, the right lower third molar [n 48] was used.

To assess reliability, 30 randomly selected OPTs were examined by two authors [CDS and AT], and 30 randomly selected OPTs were assessed twice by the first author, with a one month interval between the two observations. Intra and inter-observer agreement was determined using the Cohen’s kappa test. A descriptive analysis of the stages of visualization of the periodontal ligament according with age was done, and correlation between age and stage of visualization of the periodontal ligament was performed using Spearman rank order correlation [rho]. The level of significance was defined at *p*<0.01.

Microsoft Excel [Microsoft Corp., Redmond, WA, USA] was used for data registration and IBM SPSS Statistics 20 [SPSS Inc., Chicago, IL, USA] was used for statistical analyses.

This study was submitted and approved by the Ethical Board of the Faculty of Dental Medicine of University of Porto.

## Results

Repeated scoring of 30 radiographs revealed good agreement in both cases [k>0.80], meaning that this methodology has both good reproducibility and repeatability. Descriptive analyses of the different stages of visualization of the periodontal ligament according with age can be seen in  Table 2.

**Table 2:**
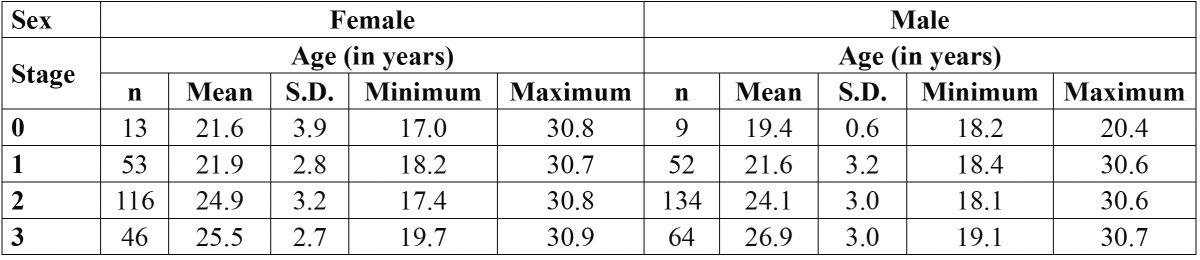
Descriptive analyses of the different stages of visualization of the periodontal ligament according with age groups (S.D.= Standard deviation).

Stage 0 first appeared at age 18 and 17 years, for males and females, respectively. Stage 1 was first attained at age 18 years for males, and 18 years for females. Stage 2 was achieved first at age 17 for females and age 18in males. The earliest appearance of stage 3 happened at age 19 years for males and females.

Females younger than 18 were classified as stage 0 [n=3, 75%] or stage 2 [n=1, 25%]. No male younger than 18 had complete apical closure. Stage distribution in participants older than 21 years of age is displayed in  Table 3.

**Table 3:**
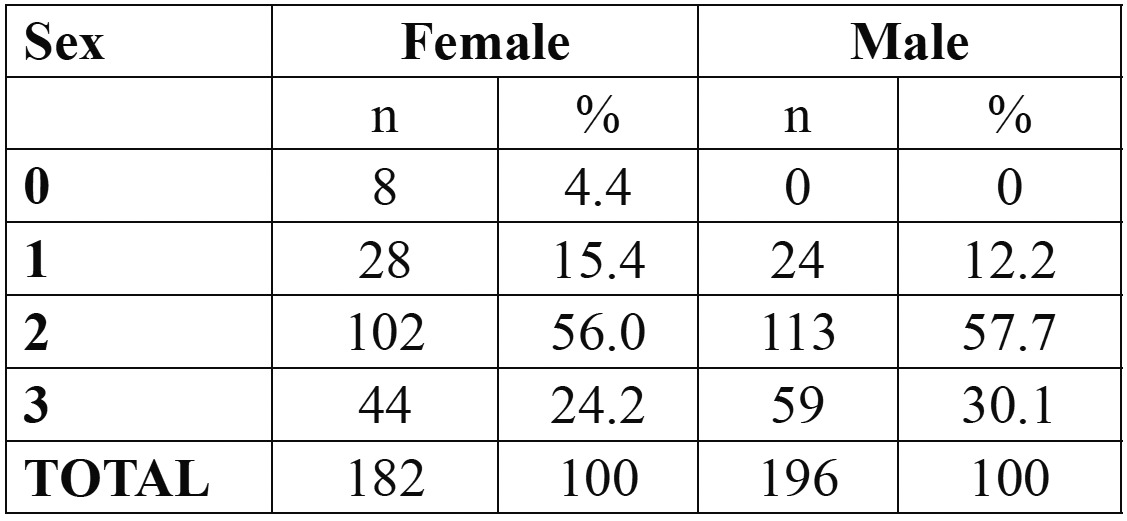
Stage distribution, by sex, in participants older than 21 years.

Considering the 21 years of age threshold, stage attainment was analyzed and results can be observed in  Table 4. Spearman rho correlation was performed to assess the strength and direction of the linear relationship between periodontal ligament stage and chronological age; it was found that there was a strong positive correlation between the two variables, for both genders [Spearman rho=0.400, *p*<0.001 and Spearman rho=0.607, *p*<0.001, for males and females, respectively].

**Table 4:**
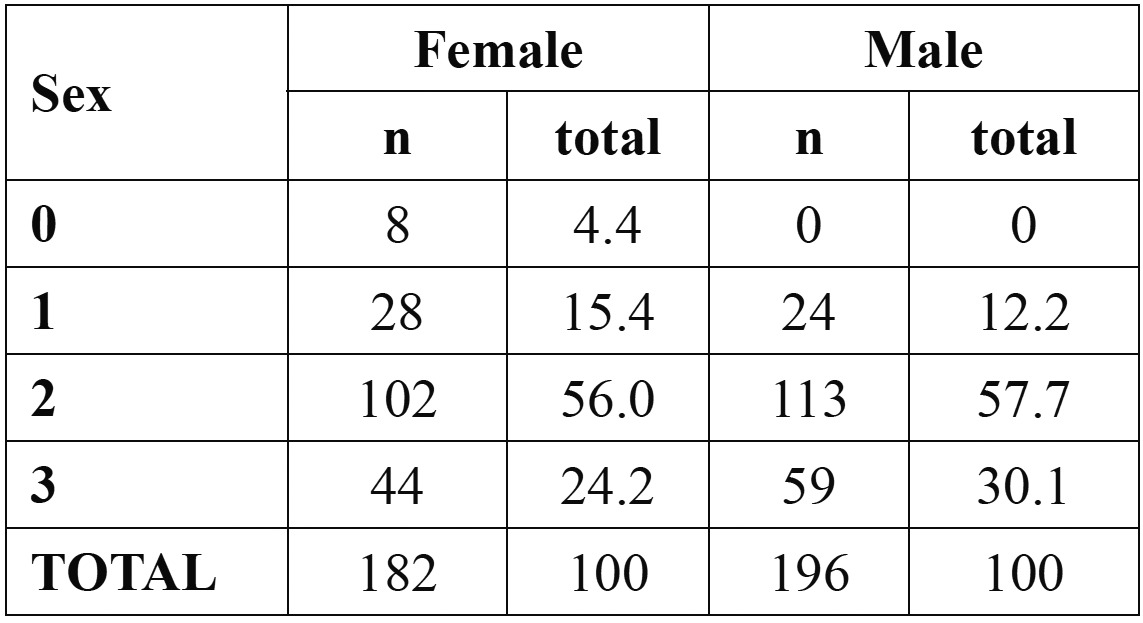
Distribution, by sex, by stage according with stage attainment.

## Discussion

Due to challenges arising, mainly, from globalization, forensic age estimation has become increasingly important. This importance is critical in some particular ages such as reaching age 14, 16, 18 or 21 years-old. For age 16 [and 18, for some populations], there are well established methodologies that can be used for this diagnosis, that rely mainly on third molar mineralization. However, this is often completed around age 20. Hence, the development of new methodologies is of the utmost importance.

AGFAD has proposed that when skeletal development of the hand is completed, an examination of the clavicles [X-ray examination or CT scan] should be performed (4). According with Schmeling *et al.* (7) and Kellinghaus *et al.* (10) clavicles examination is useful to prove that an individual has attained 21 years of age. Other authors, however, referred that this methodology is useful only until age 19 (9). Other problems associated with clavicles examination relate to difficulties to reliably determine the ossification stage of the medial clavicular epiphyseal cartilage based on conventional posteroanterior radiographic images due to the superimposition of other structures and discrepancies in staging between cases assessed by conventional radiography and CT (19). One advantage of clavicles examination is that, as it happens with other skeletal maturation methods, time-related differences are not affected by ethnicity, allowing X-ray standards for forensic age estimation to be applied ethnic groups which differ from the reference population; however, the rate of ossification is primarily affected by the socio-economic development of the population concerned, meaning that the application of X-ray standards to an accused person of a socio-economic status lower than that of the reference population usually leads to underestimation of that person’s age (20).

Periodontal ligament visualization is a methodology proposed by Olze *et al.* (3) that is based on an optical phenomenon: the disappearance of the periodontal ligament; according with the authors, the biological explanation for this maybe that, with time, the membrane becomes so narrow that it can’t be seen on radiographs. The main advantage of this methodology is, perhaps, that it relies on an OPT observation. As stated before, an analysis of an OPT is one of the main phases of forensic age estimation. Therefore, by using the same x-ray exam, time and money can be saved, and there’s no need to expose individuals to more radiation.

The present investigation showed no intra- or inter-observer significant differences, underlining the reliability of this methodology. It was also found a strong positive correlation between age and periodontal ligament staging, both for males and females [r=0.541, *p*<0.001], meaning that, as seen by other authors (3), as age increases so does the LPD staging. There were, however, some limitations in this study associated with the sample and the variables. On one hand, the population studied is limited to a single ethic group; many authors have referred that dental standards must be population specific (12,13). Therefore, these data concern only the Portuguese population, and for other populations, specific-population data should be used. Additionally, this technique requires that third molars have complete root formation, no caries, no endodontic treatment and they could not be impacted. These factors do limit the use of this technique. Nevertheless, it should be pointed out that no additional exams are needed to apply this methodology. In fact, the OPT needed to assess the LPD developing stage exists already since it is required by the AGFAD. Conversely, x-ray or CT scan of the clavicles must always be made if one wants to study the medial clavicular epiphyseal cartilage. So, even that this methodology cannot be applied to every single case, it may clear things out in some situations, avoiding more x-ray rc CT scans performance.

Olze *et al.* (3) stated that stage 2 could be used for predicting age 21 years, since all individuals in stage 2 were over 21 years. Our results are not in accordance with this statement since in our sample, 17.6% % of male subjects [n=20] and 26% of females subjects [n=14] were younger than 21 years and displayed stage 2 of periodontal ligament visualization. Stage 3, however, can better serve this purpose, particularly in male, with only 3.1% displaying stage 3 being younger than 21 years of age; in females, some improvement was found [with 15.4% [n=2] of the girls younger than 21 exhibting stage 3], but, still, it can be regarded only as a fair marker, and therefore not suitable for forensic purposes.

Thus, periodontal ligament visualization of lower third molars may become an important methodology in forensic age estimation. In this population, stage 3 can be used to state that a male person is over 21 years-old; for females, another marker should be used. Differences between studies are evident, suggesting that specific population standards should be used when applying this technique.
